# Artificial Intelligence in IVF Laboratories: Elevating Outcomes Through Precision and Efficiency

**DOI:** 10.3390/biology13120988

**Published:** 2024-11-28

**Authors:** Yaling Hew, Duygu Kutuk, Tuba Duzcu, Yagmur Ergun, Murat Basar

**Affiliations:** 1 Valley Health Fertility Center, Paramus, NJ 07652, USA; madelinehew@gmail.com; 2 Bahceci Health Group, Umut IVF Center, Altunizade, Istanbul 34394, Turkey; duygukutukk@gmail.com; 3 Department of Health Management, School of Health Sciences, Istanbul Medipol University, Istanbul 34815, Turkey; tduzcu@medipol.edu.tr; 4 IVIRMA Global Research Alliance, IVIRMA New Jersey, Marlton, NJ 07920, USA; yergun@ivirma.com; 5 Department of Obstetrics, Gynecology, and Reproductive Sciences, Yale School of Medicine, New Haven, CT 06510, USA; 6 Yale Fertility Center, Orange, CT 06477, USA

**Keywords:** artificial intelligence, quality control, quality assurance, IVF laboratory, outcomes

## Abstract

Incorporating artificial intelligence (AI) into in vitro fertilization (IVF) laboratories signifies a significant advancement in reproductive medicine. AI technologies, such as neural networks, deep learning, and machine learning, promise to enhance quality control (QC) and quality assurance (QA) through increased accuracy, consistency, and operational efficiency. This comprehensive review examines the effects of AI on IVF laboratories, focusing on its role in automating processes such as embryo and sperm selection, optimizing clinical outcomes, and reducing human error. AI’s data analysis and pattern recognition capabilities offer valuable predictive insights, enhancing personalized treatment plans and increasing success rates in fertility treatments. However, integrating AI also brings ethical, regulatory, and societal challenges, including concerns about data security, algorithmic bias, and the human–machine interface in clinical decision-making. Through an in-depth examination of current case studies, advancements, and future directions, this manuscript highlights how AI can revolutionize IVF by standardizing processes, improving patient outcomes, and advancing the precision of reproductive medicine. It underscores the necessity of ongoing research and ethical oversight to ensure fair and transparent applications in this sensitive field, assuring the responsible use of AI in reproductive medicine.

## Simple Summary

Integrating artificial intelligence (AI) in in vitro fertilization (IVF) laboratories represents a significant advancement in reproductive medicine. AI tools such as machine learning and deep learning enhance quality control, improve accuracy, and increase efficiency in tasks like embryo and sperm selection. By automating traditionally manual processes, AI reduces human error and variability, thus supporting higher success rates for IVF treatments. However, the application of AI in this sensitive area also raises ethical and regulatory challenges, including concerns about data privacy and transparency in algorithm-driven decisions. Overall, AI has the potential to revolutionize IVF by optimizing outcomes, but it requires careful management to ensure ethical standards are met and patient trust is maintained.

## 1. Introduction

The journey of in vitro fertilization (IVF) has been a scientific marvel and profound human impact, tracing back to the first successful procedure in 1978. In recent years, IVF has emerged as a symbol of hope for countless couples worldwide, serving as a pathway to parenthood when natural conception is unattainable. While the technology has advanced, pregnancy rates per cycle have plateaued. Regardless, IVF treatments carry a beacon of hope for millions of couples worldwide, offering parenthoods to many yearning for better odds. Despite the significant progress in assisted reproductive technologies (ARTs), IVF clinics still struggle with the complex interplay of factors that underlie the varying success rates.

Within this context of potential and challenge, the role of quality control (QC) and quality assurance (QA) in IVF laboratories are fundamental. These frameworks ensure precise execution of every stage of IVF, enhancing the chance of a successful pregnancy.

**Figure 1:**
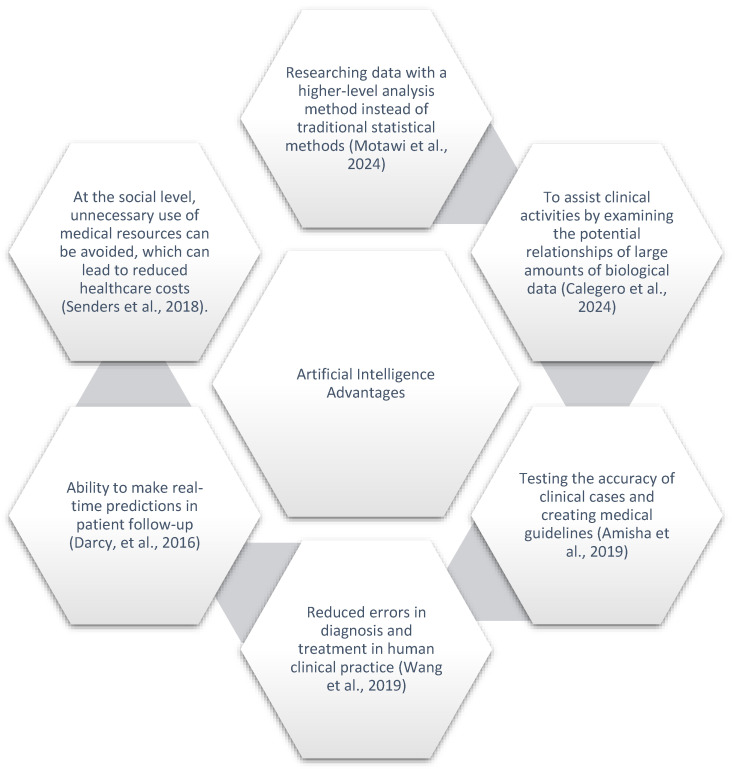
Impact of AI on medicine.

Artificial intelligence (AI) has significantly impacted medicine, demonstrating the potential to revolutionize patient care, diagnostics, and treatment planning (Figure 1).

In the world of IVF, AI, with its advanced capabilities in pattern recognition and data processing, is cautious about addressing the enduring challenges of embryo selection and genetic screening. Neural networks and deep learning, the vanguards of AI, offer an extraordinary vision for analyzing vast datasets, providing insights into the slightest distinctions of embryo development.

Dimitriadis et al. emphasize AI’s transformative role in managing “big data” for IVF. AI confers objectivity and precision in embryo evaluation, a pivotal process that has traditionally been subject to significant variability and subjective interpretation. This move towards standardization in embryo assessment and selection enhances the odds of successful IVF outcomes [1].

The launch of AI in IVF clinics signifies a new era of efficiency and accuracy. Neural networks mirror the human brain’s analytical capacity, principally in interpreting complex data from time-lapse systems. Thus, they facilitate more informed embryo selection decisions. Chavez et al. affirm the potential of AI to reduce technician variability, elevating the accuracy of critical laboratory functions, including sperm analysis and embryo selection [2].

Recent advancements in AI are redefining the landscape of IVF. Time-lapse microscopy and predictive models offer more dynamic and precise monitoring of embryo development, thereby informing real-time decisions that could significantly impact the success rates of embryo implantation and subsequent pregnancy [3].

This review navigates the promising intersection of AI and IVF, a convergence where technology is poised to refine current practices and reimagine the future of reproductive medicine. Within this context, we explore how AI’s integration with neural networks and deep learning heralds a new chapter for IVF laboratories, where the precision of reproductive treatments is vastly improved, exemplifying the hope for better success rates and the joy of life they bring to aspiring families.

## 2. AI Technologies in Medicine

### 2.1. Neural Networks and Deep Learning

Neural networks are computing systems that imitate the structure of the human brain. They enable machines to recognize patterns and solve problems like human cognition. Neural networks are used in deep learning to analyze large datasets, finding patterns and structures to make informed decisions. This is particularly relevant in medicine, where deep learning has been instrumental in advancements across various domains, including diagnostics, treatment efficacy, and rehabilitation.

### 2.2. Application in Medical Diagnostic

Deep learning has significantly enhanced a crucial medical diagnostics aspect: image analysis. In analyzing images from MRIs, CT, PET scans, and X-rays, convolutional neural networks (CNNs) have become the standard. CNNs speed up the process and improve the accuracy of diagnoses, enabling faster patient throughput, which is crucial in high-volume healthcare settings. CNN-based ultrasonography achieved comparable and greater diagnostic accuracies in lesion detection than the traditional method [4]. Uninterrupted monitoring of targeted health conditions is one prominent feature AI offers that helps pick up early signs of disease. AI is a medical diagnostic tool seen most prevalently in radiology and dermatology. In the case of melanoma screening, AI, in conjunction with CNNs, has achieved comparable competency in diagnosing and classifying images based on the presence of cancerous tissue [5].

From an interview of seventeen dermatologist professionals, the most common consensus is that AI diagnostic features are their “colleagues” or “residents” associated with their mastery levels to complement human judgment [5]. Augmented by AI, the generated reasoning can either affirm clinician judgment or fill in the gap with the overlooked details. It is deemed necessary that humans still spearhead the final call of diagnosis and communicate and interpret the results to patients. Aside from image analysis, AI can be used in speech and audio recognition. An automated speech recognition (ASR) system was explored to help transcribe conversations with patients and distinguish language patterns in which patients phrase their sentences in the psychotherapy discourse. This showed the potential to aid in future medical diagnostics and generalization to diverse clinical cases [6].

### 2.3. Advancements in Image Analysis

The ability of AI to recognize, categorize, and enumerate patterns in clinical images has transformed diagnostics. For instance, nowadays, AI systems are superior to human eyes in detecting anomalies in imaging, such as subtle changes that signify early stages of diseases like cancer or neurodegenerative disorders and their progression. This capability extends into areas requiring precise analysis, such as oncology, pathology, dermatology, and, recently, ophthalmology, providing consistency and precision in previously unattainable fields [7].

The two promising features, image segmentation and quantification, that AI has contributed to the medical field profoundly augment computer vision analysis and lead to improved treatment outcomes. From measuring the margin size of tumors (breast/liver cancer), organ delineation/localization (brain/lung imaging), surgical navigation, to retinal image analysis (diabetic retinopathy (DR), retinopathy of prematurity (RoP), and age-related macular degeneration (AMD)), image segmentation and quantification help deliver healthcare more suited to personal conditions with greater precision, as summarized by Luís Pinto-Coelho. CNN and other ML architectures, such as the vision transformer (ViT), generative adversarial networks (GANs), variational autoencoders (VAEs), and flow-based models, present standard algorithms used in image analysis by providing an image-to-image comparison, realistic image synthesis, synergy of image analysis, and image prioritization [7]. Overcoming the heterogeneity in multimodal AI algorithms and medical imaging modalities used across different datasets will prompt further advancement of AI-involved image analysis. In the field of ultrasonography, common obstacles that impede AI transformation include real-time representation, background noises, artifacts, image quality, user practice variety, and scarcity of unarchived datasets for training [8]. With enabled ML and DL, ultrasonography has extensively addressed some concerns listed above and enhanced the overall image quality.

The Partnership in AI-Assisted Care (PAC), an interdisciplinary collaboration of computer science and the medicine field, from Stanford University adopted AI’s image analysis ability to monitor ICU patients and seniors living alone to solve the unmet needs of caregivers to look after these vulnerable populations [9]. The AI was able to learn and recognize the target person’s motion pattern and habitual behaviors and detect any abnormal deviation in behavior changes with the advancement of image analysis in AI technology. They were illuminated by the implications that passive patient mobilities have on post-intensive care syndrome and long-term physical/cognitive functioning recoveries. In an adult ICU, continuous images of patient mobility activities and duration and several needed handlers were analyzed by the CNN architecture using an image capturing tool—computer vision technology (CVT)—that was testified to achieve reliable accuracy [10]. Another study utilized advanced image analysis to deeply analyze gait-related metrics (e.g., stride length, speed, swing time) of seniors with a single camera to capture long-term continuous video to reflect senior health conditions [10]. The CNN algorithm’s image analysis of walking activities facilitated a non-invasive diagnosis of Parkinson’s disease and other clinical implications.

### 2.4. Recent Developments and Innovations

Integrating deep learning (DL) in medicine has various innovative applications. The real-time use of AI tools to analyze medical images has become popular. It also analyzes medical images during surgery to guide surgeons by highlighting critical features and offering predictive insights on surgical outcomes. Integrating AI image analysis, three-dimensional (3D) modeling, and 3D printing opens new opportunities for surgeons to rehearse while gaining in-depth knowledge of patient-specific anatomical and physiological characteristics [7].

Additionally, DL models are progressively used for predictive analytics, such as anticipating disease progression and patient outcomes. This can significantly influence treatment plans and improve patient care outcomes [10]. The Food and Drug Administration (FDA) has approved several AI-supported wearable medical devices (e.g., smartphone-based ECG detector, Sugar. IQ glucose monitor). AI applications within the medical field can be seen in various disciplines, including cardiology, dermatology, pulmonary, endocrinology, gastroenterology, neurology, and drug discovery.

One of the most prominent examples of AI’s application is seen in contact tracing and infectious disease prediction. Enabled with NLP and deep machine learning models, BlueDot utilized AI in footprint tracing by collecting itinerary data from the International Air Transport Association (IATA) and generated a series of predictions of potential outbreaks during the COVID-19 pandemic [11]. Infectious Disease Vulnerability Index (IDVI) scores further predict the crisis management capability of countries according to demographics, health care, public health, disease dynamics, domestic/international politics, and economic attributes [12]. A study led by Chow et al. achieved at least 94% accuracy in predicting the COVID-19 transmission route via random forest (RF) and XGBoost (XGB) learning algorithms [13].

A novel AI application in collaboration with virtual reality (VR) unveils a new way of cognitive assessment, as examined by Brouwer et al. [14]. The VR simulation of shopper perception was implemented to track human eye movement and gazing behavior, and data underwent Logistic Regression and support vector machine to analyze key cognitive parameters in refixation, visual attention, work memory, and executive functioning. This integrated use of ML and VR showed promising potential for future prediction and diagnosis in identifying stroke patients from the health control population, while more thoughtful considerations are needed.

### 2.5. Overcoming Challenges

Despite the successes, there are still some concerns about enhancing the usability and trustworthiness of AI in medical diagnostics. Proposed by Orth et al., the 4P model of medicine (Predictive, Preventive, Personalized, and Participatory), as enshrined by the EU Medical Act, depicts a broad picture of ideal patient–physician, patient–technology, and physician–technology relationships [15]. Ensuring data privacy, ethical considerations, and transparent AI processes is crucial. For one thing, stakeholders like insurance companies with access to processed data may form a biased exclusion on insurers at higher health risk levels [11].

Data, serving as the foundation of AI, can easily fall victim to various levels of misinterpretation. Biased data can originate from the inappropriate selection of machine learning models, metrics for performance evaluation, classification of raw data, and compatibility of available data across different organizations [11]. From a clinical standpoint, the technological advancement led by AI implies undesired concerns like dehumanization in patient care, change in the patient–physician relationship, invalid validation from the use of inconsistent training/testing data, overfitting issues, and the call for additional training for clinicians to adapt to AI-driven medicine [16]. During such a transition, medical interpretation by physicians, instead of solely relying on analytical interpretation by AI, is placed with critical emphasis on all aspects of medicine to add in human touch and expertise that improves patient experience and well-being [15]. An in-depth knowledge of AI and flowing collaboration of such technology with healthcare workers and the public pose a significant hurdle to the future commoditization of AI in medical science [15]. Ongoing efforts are to develop effective, explainable, and fair models to ensure practitioners and patients trust these technologies. Apart from all the technical obstacles AI faces, the lack of human-like processing abilities (soft skills) such as a sense of humor, rationality, creativity, aesthetics, emotional intelligence, and empathy in the AI world poses a more complicated challenge [9].

In conclusion, applying neural networks and deep learning in medical image analysis represents a significant breakthrough in healthcare technology. The potential of AI technologies to revolutionize patient diagnostics and treatment and set new standards in personalized care and operational efficiency in healthcare facilities worldwide is unprecedented.

## 3. Implementation of AI in IVF Laboratories

### 3.1. Current Challenges in IVF Labs

IVF laboratories face various challenges that can affect the success rates of fertility treatments. Multiple variabilities resulting from inter-operators and inter-laboratories best reflect fluctuating clinical results. In the traditional IVF lab setting, most tasks that entail administrative, clinical, and even research decisions rely heavily on manual intervention. The staffing shortage in the growing IVF business accumulates burdens on clinical outcomes and overall patient experience. Embryo assessment techniques vary greatly, sperm and egg selection are highly subjective, selection criteria remain contentious and inconsistent across the globe, and monitoring embryo development is labor-intensive. The success of IVF treatments depends on precise, repeatable, and standardized processes. Integrating AI use into IVF labs undoubtedly offers a potential solution to shed light on those concerns.

As AI constantly grows and expands its practicalities in the vast IVF world, many concerns aroused thus lead to “AI anxiety”. Some of the most discussed AI anxiety topics in IVF are as follows: privacy violation, ethics violation, regulatory and compliance issues, costs, accessibility, patient perception, the patient–clinician relationship, job replacement, bias behavior, interpretability, explainability, and transparency. The circulated concept of clinical decision support systems (CDSSs) aside, practitioners will offer a more seamless transition to automation with human experience interplay in the final decision-making process [17].

The interpretability of the information derived from AI, commonly regarded as a “black box”, gives rise to public concerns about its epistemic and ethical consequences and further highlights the importance of interpretable ML under the subcategory of the AI program [18]. Interpretable ML-programmed AI can overcome manipulative inter-laboratory varieties in environmental conditions, culture systems, media type, patient characteristics, ovarian stimulation regimen, and protocols with its flexibility in being adjusted from the domain setting [18,19]. If users have limited knowledge of AI’s reasoning process that leads to the output, then error identification and system calibration at times of AI malfunctions would become impossible. Alternatively, should the data that ML was trained on contain bias in any form, the progressing AI development will pick up the wrong signal and make false connections of the resulting outcomes to unfaithful predictors in a magnified matter. Often neglected or intentionally, users may feed input in the hope that some correlations can be found within those specified metrics, which could seriously undermine the integrity of data interpretation from AI. The ability to translate enormous raw databases into meaningful knowledge of practical use and generalize its applications on randomized control trials (RCTs) with consistent results remains a primary challenge to AI’s implementation in the lab [18].

### 3.2. Role of AI Technologies

Implementing AI in IVF laboratories improves accuracy and consistency by helping overcome various challenges and automating complicated and subjective processes. Chow et al. claimed that by improving sperm and oocyte selection and optimizing IVF treatment regimens, AI could improve IVF outcomes. Additionally, AI can provide objective data analysis from sperm, oocytes, and embryos, leading to better decision-making [20]. A reflection on Antoine Lavoisier’s statement, “Nothing is lost, nothing is created, everything changes”, AI helps process massive data within a shorter timeframe, drastically boosting operational productivity so humans can do something more meaningful [21]. “AI is all about perception and detection, and it is empowered by accelerated computing ability, simulation, and graphic processing units (GPUs)”, says Jensen Huang, the CEO of NVIDIA, during the interview. Although AI’s implementation in IVF labs has led to many debates, AI indeed has enlightened more people to reimagine the blueprint of the future IVF world.

### 3.3. Automating Embryo Selection

Recently, one of the most popular applications of AI in IVF laboratories has been the automation of embryo selection. Nowadays, with the help of AI-driven systems, assessing embryo viability is more accurate by utilizing advanced imaging and machine learning algorithms. As Dimitriadis et al. point out, AI systems equipped with machine learning provide a non-invasive method to predict embryo quality, thereby improving the selection process by reducing the variability introduced by technicians [1]. Multivariate variables at inter-laboratory and inter-operator levels, such as the dictated time of the day for embryo quality checks, predisposed environmental conditions (altitude, humidity, vibration, etc.), static- or video-based evaluation, and patient population, can all contribute to confounder biases in the ML model, which affects the overall precision and effectiveness in automating the embryo selection process. The migration from using a “black box” (uninterpretable ML) toward a “glass box” (interpretable ML) marks a significant milestone in the improved transparency, explainability, and interpretability of AI’s implementation in embryology [19]. In place of subjective and manual evaluation of embryo qualities with various grading systems and genetic testing programs, AI can combine embryo culture, grading, and ploidy detection in one platform.

### 3.4. Data Integration and Standardization

Accessing historical data via existing laboratory information management systems (LIMS) is critical for AI model training. Provost et al. emphasize the importance of broad data analysis in AI applications, emphasizing the need for standardization and precision in clinical parameters, which AI can facilitate [22]. AI aids in analyzing the often-complex cause–effect relationship unique to the reproductive medicine field using a multivariate consideration tactic to handle data with calculated weighing coefficients that determine the priorities of each independent variable [19]. With extended learning capacities, AI is trained to process enormous amounts of data in one of the three frameworks depending on the nature of the input and desired output: supervised, unsupervised, and reinforcement learning [17]. Along with those embedded learning features and large language model (LLM) processors, AI extends its tentacles broadly to reach a wide range of data captured visually, numerically, and verbally.

### 3.5. Overcoming Implementation Hurdles

**Figure 2:**
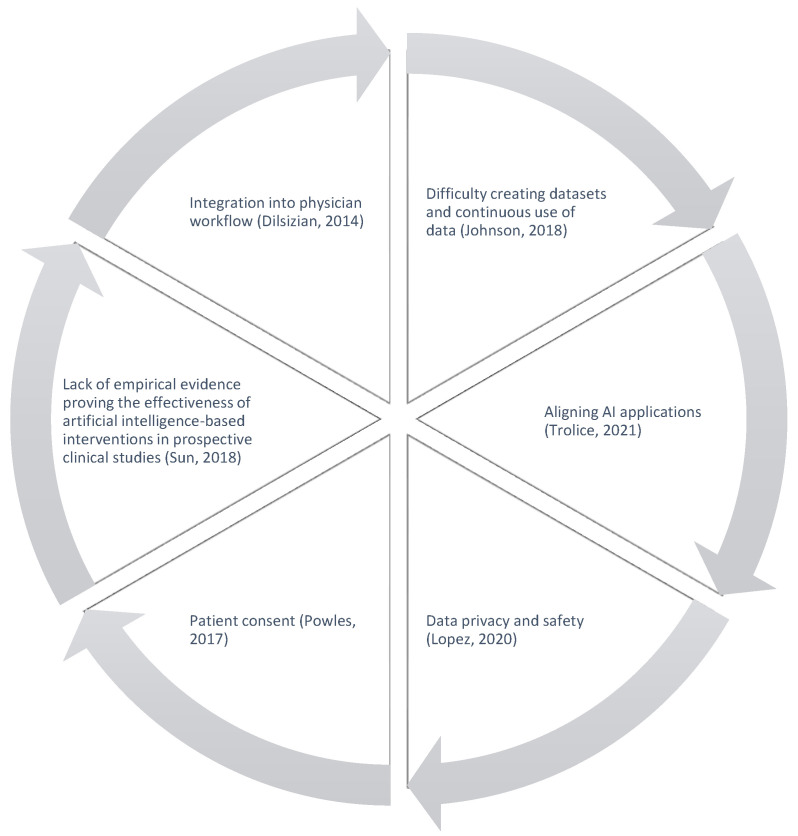
Challenges in AI.

Implementing AI in IVF laboratories has various challenges that must be addressed (Figure 2). Since the inception of AI development in the 1950s, attention has been drawn to AI’s regulatory frameworks and liability issues. However, due to the rapidly evolving pace within the AI industry, hardly and rarely have any regulatory bodies been able to conceive comprehensive legislation encompassing all applications and all periods that AI has extended into. On the global scheme, ISO 42001 and WHO have set general guidelines for managing AI gearing toward purpose focused and its ethical implications [23]. Nonetheless, compared to the speed of current AI development and deployment in the commercial market, there is minimal regulation with an actual executive effect to enforce and oversee the AI industry. Data security has come to the surface when massive sensitive data points are collected without a robust and strict outline, refining how the data are utilized and protected and if the data are funneled through the designated intention of use behind the scenes [24].

Amid the transition period to automation systems, the role of embryologists in IVF labs is even more emphasized. Upon technological maturity, AI is hoped to take over most of the IVF lab tasks, leaving only specific procedures that entail higher complexity, like ICSI, embryo biopsies, and vitrification, to manual manipulation. In addition to their technical responsibilities, embryologists are expected to dedicate more time and effort to intellectual advancement in statistics, data management, and patient communication [25].

With AI in-house, embryologists become more the observers who guard the last defense in the IVF lab, ensuring the operation of AI meets expectations. A proper check and balance system is needed to inspect the efficacy and efficiency of AI performance. A carefully designed AI model should ideally go through these three stages to achieve acceptable maturity in real-world practices: “train” itself to be equipped with the knowledge, “validate” itself to the set range, and “test” its performance on trial cases [17]. With increasing advocacy in using “glass-box” interpretable ML-integrated AI systems, the human role in all AI-involved processes becomes unalienated. It is still questionable whether the implementation of AI will eventually cut down the labor force and costs or the opposite.

AI implementation in IVF labs offers more standardized, objective, and efficient processes. This technology has the potential to enhance the accuracy of procedures like embryo selection and help manage the data-intensive nature of IVF treatments, ultimately improving fertility treatment success rates.

While the complete replacement of hands-on skills by AI is a hypothetical scenario that raises concerns about the future role of embryologists, AI will more likely augment rather than entirely replace human expertise. Embryologists will continue to play a crucial role in IVF and personalized medicine, albeit with a different focus and skill set that adapts to technological advancements and evolving patient needs.

## 4. Benefits of AI-Based QC and QA in IVF Laboratories

### 4.1. Enhanced Accuracy and Consistency

AI technologies can improve the precision and reliability of procedures carried out in IVF laboratories. With enhanced machine learning, AI is being trained to understand the structure of gametes and embryos through image analysis and is gradually becoming capable of discerning structural variances and indicating ideal locations for penetration with minimal interference to embryo developmental potential. Embryologists performing complex micromanipulation procedures such as ICSI, laser-assisted hatching, and biopsy could use AI’s strength to improve procedural precision and effectiveness [26]. AI systems reduce human error and variability by applying advanced algorithms for embryo selection and assessment and overcoming inconsistencies in embryo selection. Provost et al. reported that AI’s ability to analyze “big data” allows for more objective, standardized, and precise embryo evaluations, significantly improving the selection process for viable embryos [22]. The lack of uniformity in embryo selection criteria often gives rise to misjudgment and the waste of viable embryos with true reproductive potential. Another benefit of incorporating AI lies in its application for patient ID traceability. Extending the functions of the electronic witnessing system, the deep convolutional neural network helps achieve non-invasive “cell tracking” by countless identifiable markers seen in embryo images in the hustle and bustle of the IVF workplace [2,26].

### 4.2. Improved Outcomes

The goal of integrating AI into IVF laboratories is to improve the outcome of ART treatments. Chow et al. discuss the potential of AI to enhance fertility outcomes by improving the selection and handling of eggs and sperm and formulating more effective IVF treatment regimens. By integrating AI-based technologies, clinics can optimize the entire IVF cycle, from hormonal stimulation to embryo transfer, leading to potentially higher pregnancy and live birth rates [20]. As the “third eye”, in addition to embryologists, AI can identify subtle variances in microscopic images and motion patterns [26]. One critical function of AI is its ability to sort out relationships and patterns within many possible factors. The lab key performance indicator (KPI) is a force to be reckoned with within the IVF lab, striving for the highest standard of patient care and clinical excellence. By comparing the benchmark AI set, personal and lab overall performance are subjected to less biased evaluation and feedback from a fair, third-party “AI”. The inclusion of AI use in lab procedures allows lab staff to focus energy on other essential tasks of higher complexity and intellectual manner, like research projects and training, which ultimately benefits clinical performance and outcomes [24].

### 4.3. Scalability and Efficiency

AI systems increase efficiency and precision by automating time-consuming tasks in the IVF laboratory. Dimitriadis et al. reported that AI models are superior in reducing manual errors and minimizing staff workload by handling routine IVF laboratory tasks. This accelerates the laboratory process and allows for handling more cases without compromising quality [1]. The evolving trend in the IVF industry lies in automating tasks that are commonly deemed fundamental and repetitive but time-consuming; examples may include daily lab quality control activities and generating statistical reports. Emerging technologies such as weight-based liquid nitrogen detectors, air quality analysis, time-lapse incubators, cryo-tank storage inventory, lab instrument maintenance schedule prediction, and alarming systems, to name just a few, showcase potential applications in which automation shares partial burdens from lab staff’s daily work plates [20]. Instead of relying on momentary examination by the human eye at specific periods of the day, AI can help collect continuous data points and pinpoint outliers. With many enclosed incubation and long-term storage systems in the IVF lab, computer-based AI can minimize unnecessary disturbance and avoid introducing contaminants from these daily activities. Another primary application of AI is the processing of lab statistics. Many labs nowadays still heavily rely on physical paperwork and manual data entry. Extensive data input and possible human error affect the accuracy of data presentation. In the newly designed, integrated statistic processing channel, input values are internalized for various comparisons of the lab choice and interpreted into helpful information of desire. With the aid of AI, higher precision and efficiency in data calculation, parameter monitoring, and risk identification can be achieved to enhance the overall integrity of quality management systems.

### 4.4. Predictive Insights

AI models offer predictive insights that are crucial for personalized medicine. By analyzing extensive datasets, including historical outcomes and real-time data from current treatments, AI systems can forecast the success of specific treatment protocols for individual patients. From fertility diagnosis, treatment planning, medication dosage prescription, drug toxicity, cycle progress monitoring, and controlled ovarian stimulation protocol to adversarial events during pregnancy, AI guides clinicians with predictive insights to customize patient care accordingly [17,24]. This ability to predict reproductive outcomes helps clinicians tailor treatments to individual needs, thereby enhancing the personalization of care and improving success rates. Oocytes are deemed to contribute most to embryo development before embryo genome activation (EGA) kicks in at a later cleavage stage. Yanez et al. thus trained a support vector machine (SVM) classifier to predict embryo viability based on oocyte morphologies and early zygote mechanical characteristics at the 2PN fertilization state (including RNA gene expression, DNA repair and cellular stress response, cortical granule release, and zona hardening) and derived more than 90% precision, 95% specificity, and 75% sensitivity [27]. Additional considerations for many variables, such as patient characteristics and the big data analyzed from the more significant population, give a more accurate predictive value of the clinical outcomes. For instance, the area under the curve (AUC) of the targeting receivers provides predictive insights to clinicians and patients regarding implantation and clinical success with incrementing predictive values when more variables are accounted for [20].

However, there are concerns about over-reliance on AI and automation, such as the risk that physicians might rely too heavily on automated systems without critically evaluating their outputs. This could lead to missed nuances or individual patient factors not captured by algorithms. In addition to that, embryologists may lose some of their hands-on skills if automation handles routine tasks. This could impact their ability to adapt to unique cases or unexpected challenges. To mitigate these risks and maximize the benefits of automation in personalized medicine for IVF, it is crucial to ensure that automated systems are used as tools to support decision-making rather than replace human expertise, keep physicians and embryologists updated with training on new technologies, and ensure they are equipped to handle both the automated and manual aspects of their work and prioritize patient needs and preferences in treatment decisions. AI is only required to enhance patient care and outcomes rather than dictate them.

### 4.5. Reducing Subjectivity in Embryo Selection

The subjectivity involved in embryo selection is one of the significant challenges in ensuring consistency in IVF outcomes. AI-based tools use objective criteria derived from vast datasets to assess embryo quality, which can reduce biases and improve the decision-making process. This technological advantage ensures that the best possible embryos are selected for implantation, increasing the likelihood of a successful pregnancy.

In conclusion, incorporating AI-based quality control and assurance in IVF laboratories represents a transformative advancement in reproductive technology. With benefits ranging from improved accuracy and efficiency to enhanced predictive insights and reduced subjectivity, AI technologies are set to improve IVF treatments’ consistency and success rates significantly. As these technologies continue to evolve, they promise to deliver even greater precision and personalization in fertility care, aligning with the broader goals of modern medicine.

## 5. Case Studies and Current Implementations

### 5.1. Real-World Examples

Integrating AI in IVF laboratories has led to noteworthy improvements in various aspects of the IVF process. Here are some critical case studies that illustrate the practical benefits of AI applications in reproductive medicine:

### 5.2. Sperm Analysis and Selection

Male infertility represents a significant global problem due to its major health, social, and economic impacts [28] and leading up to 50% of infertility diagnoses [29]. With the increasing demand for fertility treatments and rising male factor infertility, enhanced efficiency, effectiveness, and precision in andrology procedures are of utmost significance to improve the current IVF lab practices and fluctuating success rates [30]. The use of AI in semen analysis has an important application in sperm selection in assisted reproductive techniques (ARTs). Images obtained from semen analysis with AI can provide embryologists with a useful tool to create time-efficient, standardized, and reliable sperm selection method, improving embryo and pregnancy outcomes [31]. Like all AI algorithms, the performance and reliability of these systems largely depend on the orderliness and completeness of the data. The abundance of sperm imaging data and collaboration between laboratories will contribute to the development of embryo selection systems [32].

The role of automated semen evaluation is even more exacerbated among patients with idiopathic diagnosis [30]. This is the point where AI comes into place and provides solutions aiding in the human decision-making process with improved GPU embedded analyzers [20]. Computer-assisted semen analysis (CASA) has dominated the mainstream of digital semen analysis in ARTs in the last decade. With its limitations on individual sperm analysis and sperm morphology assessment, there is an urge for a more comprehensive and less invasive AI-supported sperm selection technique that discloses more insightful perspectives on predicting outcomes of sperm utilization [17].

In recent years, AI has begun extending its tentacles into a wide array of vital sperm parameter evaluations including seminal fluid pH, DNA integrity, capacitation potential, morphology, and chromosomal abnormality adapting AI optical microscopic-based technology LensHooke™ X1 PRO, manufactured in Taichung City, Taiwan), comet assay, partially spatially coherent digital holographic microscope [29,30,33,34,35,36,37]. Additionally, AI serves as a preliminary diagnostic tool providing insights into male infertility by examining seminal fluid biomarker composition, presence of varicocele, hormonal imbalance, etc. [17,30]. A study that investigated AI’s prediction accuracy on testosterone deficiency syndrome (TDS), which is commonly associated with hypogonadism and type 2 diabetes, saw a ray of hope with AI’s potential to predict TDS based on ten accessible low-cost classifiers, though there exist some limitations [38]. Beyond these intrinsic factors, extrinsic conditions such as patient demographic characteristics, lifestyle, and environmental stressors complicate the interpretation of sperm analysis and influence sperm selection, ultimately by the guidance of AI through artificial neural network (ANN) learning [17,39,40].

Commercial vendors and scholars have achieved several advancements incorporating AI into sperm analysis and selection to date. Automated sperm selection technology, the single-sperm selection software (SiD), is one example of AI technology applied to individual sperm analysis and selection for intra-cytoplasmic sperm injection (ICSI). The use of SiD with its selection criteria focuses mainly on sperm motility and progression before ICSI. It has proven to achieve competent KPIs as defined in the Vienna Consensus in juxtaposition with human judgment [41]. The morphokinetics of the zygotes rooted in the SiD cohort are similar, and the overall quality indicators even slightly outperform the traditional sperm selection process conducted manually by embryologists [41]. Technologies such as SpermQ, which measures flagellar beats, and Mojo AISA offer automated semen analysis (concentration, progression, and motility) in a very rapid fashion, which overcomes manual oversight and time-consuming deficiencies commonly seen in CASA systems [30,34,42]. A smartphone-based semen analyzer that analyzes unprocessed semen through a microfluidic biosensing microchip device was developed for the ease and convenience of semen analysis of high accuracy in measuring sperm concentration, motility, and linear and curvilinear velocity [30,38,39]. Using a CNN learning technique, AI helped detect sperm location in microdissection testicular sperm extraction (micro-TESE) and achieve at least 84% positive predictive value from the otherwise challenging testicular non-obstructive azoospermia (NOA) cases [36,37]. The non-invasive AI viability test of immotile sperm based on nucleus morphological variances became available when a study revealed great confidence in achieving over 90% comparable high accuracy, sensitivity, and specificity to the traditional cytotoxic staining method [43]. A research group testified the precision and accuracy of classifying sperm morphology and abnormalities by head, vacuole, and acrosome labels with deep transfer learning (DTL) and deep multi-task learning (DMTL) algorithms [44]. An AI-powered sperm DNA fragmentation (SDF) test showed a greater capacity to handle more samples in less time and reduce subjectivity in comparison with manual SDF [45].

In this regard, AI shows great capacity to handle large datasets and predict lab/clinical trends/outcomes. Picking a good sperm is as equally important as having a good oocyte, although the sperm source is often more abundant and accessible from a larger pool of candidates. In the state-of-the-art IVF clinic, embryologists always strive for the best possible outcomes derived from the best gamete combination. The importance of oocyte quality from fertilization to blastocyst development is well known to most, yet people tend to underestimate or neglect the andrology processes involving sperm analysis (SA), sperm selection, and sperm preparation techniques by mistakenly simplifying the often-multi-step, thought-provoking sperm processing complexities. AI serves as a helpful tool in andrology that mitigates human errors and inter- and intra-operator variabilities, meanwhile identifying sperms with the most reproductive potential, optimizing resource (time and cost) allocation, and enhancing standardization and objectivity of sperm assessment. With the assistance of image recognition and AI, along with its features in deep learning (DL): artificial neural networks (ANNs), convolutional neural networks (CNNs), and natural language processing (NLP), reproducible clinical outcomes should offset the effects of a variety of sperm processing techniques adopted by inter-operators and inter-clinics [30,35].

In functional urology, it is seen as promising to investigate the pathophysiology of urinary system dysfunction and to improve the existing assessments such as dynamic magnetic resonance imaging or urodynamics as a diagnostic tool. The measurement records obtained through artificial intelligence can also contribute to surgical education and training. Thanks to the models to be created, innovative devices such as the e-bladder diary and electromechanical artificial urinary sphincter can be developed [46]. In a study, an artificial intelligence model was developed to predict clinically significant sperm parameters after varicocelectomy using preoperative hormonal, clinical, and semen analysis data. It has been reported that thanks to this model, it is possible to predict whether a patient with varicocele will benefit from treatment [47].

### 5.3. Embryo Selection and Evaluation

Recent case studies using AI in embryo selection demonstrate AI’s capability to improve the accuracy of selecting viable embryos for implantation. Traditional embryo evaluation involves subjective annotation and grading criteria from inter-operators and intra-laboratories. IVY, an AI-based deep learning algorithm that assesses continuous time-lapse videos instead of static images to predict fetal heartbeat corresponding to embryo quality selected by AI, may address the annotation issue [48]. The analysis of both embryo morphokinetics and omics parameters like oxidative metabolites, protein expressions, and proteomic information released in spent culture media have demonstrated insightful predictive values over implantation potentiality [25,49,50]. Yet, the real value of omics alone in evaluating embryo quality needs further validation [17]. Advanced AI algorithms analyze time-lapse images of embryos in optimal closed embryo culture systems (CECSs) since syngamy event to predict their development potential, significantly increasing the likelihood of successful pregnancies [20]. ERICA, an AI-based static image analysis technology that detects ploidy status and classifies embryo viability, shows high correspondence to spontaneous abortion outcomes [2]. Intelligent data analysis (iDA) scores, supported by a 3D CNN and fed with morphological time-lapse images, were found to be a useful tool to predict live birth and miscarriage rates with high confidence levels; patterns of iDA scores in ploidy evaluation were discovered yet require further investigation [3,48,51,52]. The AI-combined embryo evaluation method has been adopted by several fertility clinics worldwide, providing a higher success rate compared to traditional manual selection methods. One thing to bear in mind is the complexity of endometrium receptivity interplays in the embryo selection process, which should also be taken into the calculated algorithm for a more accurate prediction of implantation potential when paired with various embryo qualities [17].

The advanced imaging technology avoids photodamage and stain perturbation that commonly comes with traditional imaging systems, which immensely protects embryo integrity and enhances future usability. EVATOM—a label-free, non-invasive, optical phase imaging, and ML-based embryo viability assessment tool—offers a novel solution addressing those concerns by incorporating the advantages of the quantitative phase imaging (QPI) technique, in combination with other methods to provide information on embryo structure (cell counts and size) and composition (DNA/protein concentration) [53]. Beyond the superficial evaluation of morphology, embryo imaging in synchronization with AI has now supported a thorough investigation of zona pellucida, inner cell mass, trophectoderm, morphokinetics, collapsing and re-expansion events, and nuclei content.

### 5.4. Predictive Outcomes in IVF Treatments

AI’s predictive capabilities have been effectively used in clinics to anticipate the outcomes of IVF cycles. By analyzing past IVF attempts and patient-specific data, AI models can forecast the likelihood of success for different treatment options, allowing clinicians to tailor approaches to individual patients [54]. As mentioned earlier, AI has the potential to create a personalized treatment cycle based on biological markers, previous IVF attempts, gravidity, responses to medications, other environmental variables, etc. While determining gonadotropin dosage to cultivate multiple oocytes within a cohort, AI has shown abilities striving for the lowest but most effective follicular stimulating hormone dosage to maximize its impacts and avert ovarian hyperstimulation syndrome through ML according to patient characteristics [17]. During trigger point, AI helps analyze the underlying roots (AMH level, follicular size, ultrasound scans) that may interfere with the stimulation outcomes [17]. With extensive research on AI, sperm selection, fertilization potential, and blastocyst formation could become more predictive.

### 5.5. Research Findings

Numerous studies have validated the effectiveness of AI in enhancing IVF outcomes:

**Improvement in embryo selection:** Studies have shown that AI can increase the precision of embryo selection. Research by Yang et al. discusses how AI, through detailed analysis of embryo morphokinetics, can identify the best candidates for transfer, leading to higher implantation rates and better overall pregnancy outcomes [55]. The strengthened identification ability on trophectoderm and inner cell mass allows AI to grasp a more in-depth understanding of embryo quality and clinical outcomes. Besides embryo morphological characteristics, other factors such as age, medical history, treatment response, and so on could all contribute to pregnancy results. The study performed by Khosravi et al. discovered pregnancy probabilities in correlation with embryo qualities and patient age, 13.8% (age ≥ 41 and poor quality) to 66.3% (age < 37 and good quality), using the STORK framework to analyze and predict embryo qualities, and utilized DNN and the chi-squared automatic interaction detection (CHAID) algorithm-based decision tree method to investigate the relationship of confounders [32]. Many papers have verified the concordance rate of non-invasive/minimally invasive chromosome screening in chromosomal ploidy status detection [56,57]. Corroborated by the AI-embedded random forest (RF) algorithm in non-invasive chromosome screening (NICS-AI), data showed an improved live birth rate in the patient group of advanced maternal age and equivalent clinical outcomes in patients aged <35 when compared with the traditional morphology grading system [58].

**Enhanced fertility treatment:** AI applications become prevalent throughout fertility treatment. Medenica et al. explored AI’s role in diagnosing infertility issues, enhancing sperm analysis, and predicting treatment outcomes, and confirms that AI significantly improves treatments’ diagnostic accuracy and effectiveness in junction with ARTs [59]. An AI-supported platform, “chatbot”, has been developed for the convenience of communication for patients on issues related to fertility treatment, medication usage instructions, and concerns to ensure patient care is delivered in a timely fashion [24]. In addition, “chatbot” has made baby steps toward initial infertility assessment with enhanced word token processing capacity using large language models (LLMs) and ANNs [17].

### 5.6. Ethical and Societal Implications

AI refers to computer systems designed to think or act like humans- rational thinking and acting systems. AI shows that these systems affect not only the way people think and act but also the way healthcare professionals work and learn [60]. In healthcare institutions, AI has been reported to support workload, performance, teamwork, satisfaction, and production [60,61]. The transition and mergence of AI applications in patient daily life is predicted to accelerate as more healthcare encounter take place at home or in a remote mobile environment besides clinics [62,63]. Rising standards of living, increasing rates of chronic diseases, and the constant development of costly new treatments contribute to this trend. It is discussed whether AI can feel or express real empathy according to the human characteristics that is sculpted by social norms, ethical values, education systems and parenting [64,65,66]. Some AI technology researchers have become interested in how AI technologies can be attentive [67]. Artificial empathy refers to the coding of empathy into machines in order to feel or show empathy in users [68,69].

Debate continues when it comes to the explainability of AI in healthcare. A comprehensive assessment is required not only on technological issues but also on ethical, medical, legal, and social issues. An ethical assessment should also be made for these topics [70]. Legal and ethical uncertainties surrounding this medically criticized issue may hinder the advancement of technologies that will improve patient and public health [71]. Explainable AI has evolved as a subgenre of AI that focuses on presenting complex AI models to humans in a systematic and interpretable way. A series of workshops were held on interpretable and explainable artificial intelligence. In this way, although not all questions can be answered, it is the image of interpretable techniques that direct future development [70].

Three main areas for explainability can be identified:

- Informed consent: It is the standard for using patient data in AI applications. Consent must be obtained by stating the purpose and outline of the project [72].
- Certification and approval as medical devices (such as Food and Drug Administration, Medical Device Regulation): Although relevant organizations have lagged behind in the development and marketing of products, it has been stated that in the near future, it will be mandatory for AI-based medical device/software manufacturers to provide information about training and testing of models, data, and general development processes [72].
- Liability: It has been reported that the patient should make or reject the clinical decision together with physicians, and the legal consequences that may arise should be evaluated from a legal perspective [72].

Is explainability legally required? If necessary, the question arises as to what extent it is required. Transparency, traceability, and public administration should be provided with higher standards regarding health services and individual patient rights [54]. For the detection, prevention, and treatment of disease, attention should be paid to data privacy and security, patient consent, and autonomy. For such, adapting to technological developments is necessary by complying with all legal regulations in the first place [70].

The integration of AI in IVF also raises ethical and societal concerns, particularly regarding privacy, consent, and the implications of algorithm-driven decisions in human reproduction. The moral debate needs careful consideration, as highlighted by Tamir, who suggests that AI in IVF must be guided by bioethics and AI ethics to ensure responsible and ethical use of technology [73]. The long-term impacts of automation on IVF processes and the offspring generated remain unclear. However, AI likely promotes fertility treatment accessibility that may resolve social inequalities within diverse patient groups [74]. While seeking alternative ways of conducting IVF business, lab professionals should remain skeptical about AI’s genuine role in optimizing procedures and treatment efficiency in contrast to its visioned trajectory. It becomes a question to all fertility specialists if incorporating AI and robotics justifies the costs and purpose of serving patients in the best possible interest. IVF fertility care is a kind of medicine that shares substantive emotional burdens with patients. Maintaining a proper balance while delivering humanitarian care in conjunction with technological advancement is a topic of crucial debate [24]. Perhaps the ideal scenario where humanity and AI could be of mutual prosperity is by withholding the concept stated by Ginni Rometty, executive chairperson of IBM, “the reality is this technology will enhance us. So instead of artificial intelligence, we will augment our intelligence” [24].

In conclusion, these case studies and research findings illustrate that AI has become invaluable in IVF laboratories, improving fertility treatments’ efficiency and efficacy. However, as with all advancements, the application of AI in this sensitive domain must be handled with a robust ethical framework to ensure that these technologies benefit individuals and society.

## 6. Prospects and Challenges

### 6.1. Innovative Trends

As AI technology advances, its applications within IVF are expected to become more innovative and far-reaching. Future trends in AI for IVF include the following:

**Genetic Screening and Analysis:** AI algorithms are poised to play a significant role in genetic screening, helping to identify genetic markers that may influence the outcomes of IVF treatments. This capability could bring about highly personalized fertility treatments, where interventions are tailored to the genetic makeup of each patient, potentially increasing the success rates of IVF procedures. Comprehensive chromosome screening collects DNA molecules shed from embryos in spent culture media or blastocoel fluid with non-invasive or minimally invasive sampling methods has potential to be widely incorporated into AI soon in place of invasive biopsy methods, especially with the innovation of specialized biochips and lab-on-the-chip technology [75,76]. Recently, Gattaca Genomics announced its upcoming 3-year research project objective to investigate AI’s efficacy with time-lapse embryo scopes aiming to deliver a comprehensive analysis in all concerning subjects with a particular focus on non-invasive genetic screening and outcomes in comparison with traditional PGT testing across various continents and patient population/clinical practices. Embryo viability determined by various mosaic thresholds set in genetic testing labs can pose a limitation that further affects the embryo utilization rate. Standardization in embryo genetic testing methods by AI deep learning algorithms helps provide a more comprehensive molecular insight when dictating embryo disposition.

**Enhanced Embryo/Gamete Imaging:** With the advancements in imaging technology combined with AI, more sophisticated methods of embryo assessment are being developed. These methods will provide deeper insights into embryo viability by analyzing factors beyond what the human eye can see, improving the selection process for implantation [58]. Pursuing higher pixel resolution and optimized GPU brings a new height for image/video processing and analysis. The capacity that image-capturing systems can intake at the one-time point has escalated tremendously with speedier turnaround time, improved accuracy, and precision in the throughput.

The application of the optimized imaging system elucidates phenomena and mechanisms hardly seen in oocytes and spermatozoa by naked eyes. Beyond morphology, AI can support the evaluation of sperm cytokinetics and predict future zygote performance. Business ventures develop computer-based software systems analyzing individual sperm motility patterns in motion “as they run, collide, and switch directions.” Numerous AI-supported tools were developed to identify oocyte qualities and acquire predictive embryo qualities through empirical evidence to offer prognostic suggestions on oocyte reproductive potential. Manufactured in Canada, VIOLET^TM^, a CNN-trained oocyte imaging software, analyzes the morphology of targeted oocytes and derives possible correlation and predictive values between oocyte appearance and fertilization potential, blastocyst formation, and live birth rates [77]. A deep neural network (DNN), utilizing DeepLabV3Plus and SqueezeNet architecture, was applied toward oocyte meiotic maturity classification to differentiate M1, M2, and GV with a list of categorized morphological characteristics [78]. Oosight embedded with an enhanced high-contrast, birefringent imaging system offers a non-invasive, quantitative grading criterion for oocytes with special analysis features on zona pellucida, spindle, and oolemma that benefit procedures like intracytoplasmic injection and somatic cell nuclear transfer. OsteraTest, encoded with eight ML models, showed up to 86% accuracy in predicting embryo blastulation until day 5 based on the gene expression of oocyte cumulus cells [79]. More advanced technologies in embryo and gamete imaging are forthcoming.

Challenges to the current IVF imaging technologies in embryos, sperms, and oocytes exist. Although time-lapse machine learning has proven its effectiveness in extracting embryo images for analysis, human verification at specific intervals for each embryo developmental stage is needed to ensure accurate interpretation of data input [20]. Static image versus continuous video raises another concern about accurately depicting embryo implantation potentiality during dynamic phases of collapse and re-expansion [20]. In automated semen analysis, the limitation on the concentration threshold still needs to be addressed in many software systems. To strengthen the embryo quality predictive confidence in oocyte screening, the analytic algorithm should identify and account for peculiar morphological structures such as smooth endoplasmic reticulum, vacuoles, granulation, etc.

**Robotics:** Automation in IVF procedures has made a critical stride in remodeling IVF lab structures and improving procedural efficiencies and performance. Collaborating with hospital and university laboratories, several pioneering startups such as Overture Life, AutoIVF, IVF 2.0, Conceivable Life Sciences and Fertilis, propose innovative applications and ideas for incorporating robotic technology into current clinical and lab practices.

Sperm-injecting robots facilitate the micromanipulation process traditionally performed by experienced embryologists, which has proven effective in fertilizing actual patient oocytes, eventually leading to the first reported clinical pregnancies [76]. Despite its initial success, manual assistance during the pre-injection steps (tail immobilization, sperm loading) of the ICSI procedure still requires certain degrees of human intervention as Dr. Palermo stated. The concepts of the biochip and miniaturized lab-on-a-chip technology, with a greenhouse-like automated growth media dispensing culturing system and additional add-on features, provide the overlook for prescient applications of what capacity automation IVF could offer in the future and possibly in the space [76]. The 3D printout structure and micro-cradles standardize conventionally time- and labor-intensive deliberate procedures such as ICSI, oocyte vitrification, and stripping [76]. Another area of use for automation in IVF is the replacement of repetitive and time-consuming tasks such as dish preparation. Validated by Lattin et al., the droplet dispensed by a robot demonstrated a substantially higher blastocyst forming rate and similar pH conditions compared to manual preparation [80]. Clinically, robot-assisted platforms have assisted in various reproductive surgeries with comparable outcomes and enhanced patient experience, including endometriosis, ovarian transplantation, tubal re-anastomosis, and fertility-sparing, providing surgeons with astute guidance, control, and images [81]. The extended use of robotics in gene editing, artificial womb manipulation, sperm picking, embryo transfer, and blastocyst biopsy requires further exploration.

Robotics in the IVF lab has set forth a new era of reproductive medicine and assisted reproductive technologies. The inclusion of robotics in IVF labs benefits the work of lab professionals and business ventures by addressing the shortage of staffing, resource allocation, human error, exhaustion, quality assurance of standardized procedural processes, increased volume of fertility treatments, etc. For patients, IVF treatment will likely become much more affordable and accessible with its manufacturing-like production streamline. On the other hand, of the many promising advantages that robotics may offer, there are challenges that automation systems incur and rely on human experience. At the early stage of robotic technology in the IVF industry, rigorous verification and validation should be in place to confidently ensure the delivery of consistent performance with acceptable variations.

### 6.2. Ethical and Regulatory Considerations

The application of AI in IVF also raises significant ethical and regulatory challenges:

**Privacy and Data Security:** As AI systems require access to large volumes of sensitive personal and genetic data, ensuring the confidentiality and security of these data is paramount. Clinics and researchers must navigate the complexities of data protection laws to maintain trust and comply with legal standards. On the one hand, validating AI’s efficacy in IVF treatment inevitably requires a great deal of patient clinical data to be collected to generate a baseline performance indicator recognized universally to be further testified on more RCTs [18]. On the other hand, the platform where data interface must fortify the safety net where identifiable information of patients may be disclosed to unintended parties.

**Algorithmic Bias:** The risk of bias in AI algorithms can lead to disparities in treatment effectiveness across different populations, namely, groups classified by a series of identifiers such as age, BMI, ethnicity, predisposed genetic makeup, epigenetics, etiology, diagnosis, treatment, biomarkers, etc. [40]. Addressing these biases requires continuous refinement of AI models to ensure they are equitable and do not perpetuate existing health disparities. Moreover, there is mutual agreement that the AI algorithm is dynamic and constantly acquiring new data input. A prediction based on the developing AI-generated algorithm should be a valuable tool aiding diagnosis, treatment, and ART procedures rather than completely replacing human judgment. Such an algorithm provides general perspectives on average populations. However, other complex factors must be considered meticulously to make a more thoughtful and comprehensive clinical decision. Implementing clinical information and embryo images into one designated AI model for the best prediction analysis and tailored treatment [82] is critical. The validation of the algorithm should be regularly performed to examine AI’s overall effectiveness and efficiency in achieving refined purposes. An AI-generated algorithm’s credibility depends on its accuracy, sensitivity, and specificity [18,82].

**Consent and Transparency:** Patients must be fully informed about how AI is used in their treatment and to what extent, including the benefits and risks. This transparency is crucial for ensuring informed consent and maintaining the ethical integrity of AI applications in healthcare. Patients should be encouraged to exercise their reproductive autonomy of their free will [18,53]. Due to the limited understanding of AI’s accurate processing mechanism currently, lab staff and clinicians should educate themselves on the strengths and limitations of using AI and verify the efficacy of AI in individual labs before implementing it in real patient cases. Most AI-based tools in IVF are propelled by commercial producers nowadays with a “profit in mind” philosophy that could bring about more biases and confusion. Before obtaining solid scientific evidence backing it up, academic endeavors should continuously challenge the outpaced industrial advancement. Open data access (under circumstances where patient private information is not breached) and coding of the ML algorithm may be a good start-off point to enhance transparency among commercial developers, clinicians, embryologists, and patients [18].

### 6.3. Legal and Societal Implications

As AI integrated into IVF treatments, it will also impact legal and societal norms. Issues such as the ownership of genetic data, the implications of embryo selection based on genetic characteristics, and the role of AI in decision-making processes in reproductive medicine require thorough examination and thoughtful regulation [59]. The US executive order for the Safe, Secure, and Trustworthy Development and Use of Artificial Intelligence draws societal attention to collaboratively frame the safe boundary use of AI. Currently, to our best knowledge, there is no set of official rules or entity bodies within the US that directly regulates the use of AI in general or in the IVF lab, which raises significant concerns about its credibility when AI has started paving its way into the IVF industry, especially with rapid impel from commercial producers. Fortunately, the European Union’s General Data Protection Regulation (GDPR) covers some common grounds for implementing AI technology to ensure patients are protected from automated decisions in the European continent [18]. The EU AI Act set an excellent global example with a clear outline of various AI employment risk-level classifications. All in all, without a clear definition of what AI entails and proper regulation in place, legislative bodies encounter difficulties clarifying the liability and accountability of AI use. Until further clarification, we must ponder this question: Who should be held responsible for the adverse events when AI is involved?

Countries with strict limitations on IVF treatment procedures could become beneficiaries of AI-driven systems when thorough data analysis and precise outcome prediction are achieved to make patients and clinicians better informed and rational decisions made [17]. Ethical concerns in using AI emerge, revolving around patient safety and its long-term impacts on future offspring and imbalanced societal structure; public perception of embryo/gamete quality grading notations can be oversimplified and misinterpreted into binary classifications such as “good or bad”, which leads to behavioral favoritism against actual reproductive potential [18,30,53].

The future of AI in IVF presents a landscape of remarkable potential paired with significant challenges. The ongoing integration of AI will likely revolutionize reproductive medicine by enhancing diagnostic accuracy, treatment personalization, and overall treatment outcomes. However, the success of these technological advancements will depend on the careful management of ethical, legal, and societal implications. Balancing innovation with responsibility will be vital to harnessing AI’s full potential in improving IVF success rates while maintaining patients’ trust and safety.

## 7. Conclusions

Integrating artificial intelligence (AI) into in vitro fertilization (IVF) laboratories marks a significant advancement in reproductive technologies. By leveraging AI capabilities, including machine learning, neural networks, and deep learning, IVF clinics are poised to enhance fertility treatments’ precision, efficiency, and outcomes (Table 1).

### 7.1. Summary of Transformative Potential

AI technologies offer transformative potential for IVF laboratories in several key areas:

**Enhanced Accuracy and Consistency:** AI-driven tools provide more accurate and consistent assessments of embryos, reducing human error and variability in the selection process. This leads to improved success rates in embryo implantation and pregnancy.

**Predictive Analytics:** AI algorithms excel in analyzing vast datasets to predict treatment outcomes, helping clinicians personalize treatment plans based on predictive insights about patient responses.

**Operational Efficiency:** Automation and AI-driven processes streamline operations within IVF labs, reducing the manual burden on staff and enabling clinics to handle a higher volume of cases without compromising care quality.

**Ethical and Regulatory Adherence:** As AI applications in IVF continue to grow, developing ethical guidelines and regulatory compliance remains crucial. Privacy, data security, and informed consent are paramount to maintaining trust and integrity in using AI in reproductive medicine.

### 7.2. Empirical Support and Research

Numerous studies and real-world applications support the benefits of AI in IVF. For example, research has shown that AI can improve embryo selection accuracy, leading to higher pregnancy rates [82]. Additionally, AI’s role in genetic screening can help identify optimal embryos, further enhancing treatment outcomes.

### 7.3. The Need for Ongoing Research

Despite the promising advancements, continuous research and development are essential to address the challenges associated with AI in IVF, such as algorithmic bias and ethical concerns. Collaborative efforts among technologists, clinicians, ethicists, and regulatory bodies are necessary to develop AI tools that are not only effective but also equitable and ethical.

### 7.4. Future Directions

Looking forward, the field of IVF will likely witness further integration of AI, particularly in areas like genetic analysis and real-time monitoring of embryo development. These innovations could lead to even more personalized and precise treatments, ultimately improving the success rates of IVF procedures.

In conclusion, AI represents a pivotal development in reproductive technologies, potentially revolutionizing IVF treatments. As this technology continues to evolve, it is imperative to balance innovation with ethical considerations to fully realize AI’s benefits in enhancing the quality and success of IVF therapies. This will ensure that advancements in AI not only lead to better outcomes but also adhere to the highest standards of medical ethics and patient care. Such artificial intelligence studies reduce healthcare professionals’ workloads, increase their performance, production, and satisfaction in IVF, where teamwork is essential, and contribute to the quality of healthcare institutions by increasing patient satisfaction and healthcare service delivery. Healthcare managers need to be familiar with artificial intelligence studies, which will be used much more in the future, and to include such applications in their institutions. Such artificial intelligence studies reduce healthcare professionals’ workloads, increase their performance, production, and satisfaction in IVF, where teamwork is essential, and contribute to the quality of healthcare institutions by increasing patient satisfaction and healthcare service delivery. Healthcare managers need to be familiar with artificial intelligence studies, which will be used much more in the future, and to include such applications in their institutions.

## Figures and Tables

**Table 1:** AI technology applications and impacts.

AI Technology	Application	Impact
*Neural Network*	Pattern recognition in embryo development	Enhanced embryo quality assessment
*Deep Learning*	Analysis of embryo morphokinetics and sperm motility	Improved diagnostic accuracy in fertility treatments
*AI Image Analysis*	Improvement in IVF success rates via image-based analysis	Higher pregnancy rates and reduced variability
*Predictive Models*	Prediction of IVF treatment success and customization of protocols	More tailored and effective fertility treatments
*Automated Embryo Selection*	Reducing human error and standardizing embryo selection	Increased consistency in selecting viable embryos

## Data Availability

No new data were created or analyzed in this study. Data sharing is not applicable to this article.
